# Modified correlated measurement errors model for estimation of population mean utilizing auxiliary information

**DOI:** 10.1038/s41598-024-61609-y

**Published:** 2024-05-15

**Authors:** Housila P. Singh, Neha Garg

**Affiliations:** 1 School of Studies in Statistics, Vikram University, Ujjain, Madhya Pradesh India; 2 School of Sciences, Indira Gandhi National Open University, New Delhi, 110068 India

**Keywords:** Study variable, Population mean, Auxiliary variable, Correlated measurement errors model, Bias, Mean squared error, Applied mathematics, Statistics

## Abstract

The existence of measurement errors cannot be avoided in practice. It is a prominent fact that the existence of measurement errors diminishes conventional properties of the estimators. A modified correlated measurement errors model has been proposed. Shalabh and Tsai (Commun Stat Simul Comput 46(7):5566–5593. 10.1080/03610918.2016.1165845, 2017) correlated measurement errors model is a particular member of the suggested modified model. In this article, we have tackled the estimation of population mean utilizing auxiliary information under modified correlated measurement errors model. We have developed ratio and product estimators and studied their properties in case of simple random sampling without replacement (SRSWOR) up to first order of approximation. It has been illustrated that suggested ratio and product estimators are more efficient than the conventional unbiased estimator as well as Shalabh and Tsai (Commun Stat Simul Comput 46(7):5566–5593. 10.1080/03610918.2016.1165845, 2017) ratio and product estimators under very realistic situations. An empirical study has also been performed to demonstrate the merits of the recommended estimators over other estimators.

## Introduction

The integration of additional available information on auxiliary variables at the estimation stage in survey sampling has been thoroughly discussed. To investigate précised estimators of the population parameters of the study variable Y has attracted much attention of the survey statisticians utilizing available information on auxiliary variable X. In this context, the literature provides several procedures such as ratio, regression, product, ratio-type and product-type exponential estimators, logarithmic ratio and product-type estimators along with their ramified versions for precisely estimating the parameters under investigation.

These estimation procedures have been proposed under the supposition that the observations gathered are free from measurement error (ME). In most practical situations, this type of circumstance is not usually encountered. Generally real data includes observational errors owing to several factors, including memory failure, excessive or insufficient reporting, prestige bias, etc. The readers are different books Cochran^1^, Murthy^2^, Carroll et al.^3^, Singh^4^, Fuller^5^ and Cheng and Van Ners^6^ etc. The term measurement error is the difference between true value and observed value which influences the findings of real-world surveys. We usually assume the accuracy of all the recorded and processed data. Though it is entirely hypothetical in surveys carried out in real life. A variety of factors, such as interviewer and respondent bias along with the errors occurred during collecting and processing the data, and many more, can lead to measurement error. So, it is important to investigate the measurement error because these issues are likely to arise in any kinds of surveys.

The values of the variable are reported to have some measurement errors (MEs) regardless of detecting the actual values of the variable under consideration. Without taking the MEs into consideration, the estimates seem incomplete which misleads the inference of the study. Various authors including Shalabh^7^, Manisha and Singh^8^, Singh and Karpe^9–13^, Diana and Giordan^14^, Gupta et al.^15^, Tariq et al.^16,17^ and Singh et al.^18^ have focused on the estimation of various parameters such as population mean, total, ratio, product and variance under MEs.

In the abovesaid studies, the authors have discussed only the case of uncorrelated measurement errors (UMEs) existing in both the study and auxiliary variables. However, in practice UME situations usually do not exist. For example, usually the same survey personal collects data on study and auxiliary variables both and so it may not be reasonable to presume that the MEs in both the variables are independent. Rather, they will be dependent (i.e., correlated) and this dependence in MEs may arise due to the hidden intrinsic tendencies of the surveyor. For further illustration, readers are referred to Shalabh and Tsai^19^, pp. 5567–5568. Shalabh and Tsai^19^ were the first who discussed the impact of correlated measurement errors (CME) over the performance of ordinary ratio and product estimators of population mean. Later Boniface et al.^20^, Bhushan et al.^21,22^ and Kumar et al.^23^ have evaluated the performance of some estimators of population mean under CME.

Taking motivation from Diana and Perri^24^ and Shalabh and Tsai^19^ work, we have developed a modified correlated measurement errors model. This paper is an effort towards developing ratio and product estimators under a modified correlated MEs model.

The remaining sections of this article are organized as follows: Shalabh and Tsai^19^ correlated measurement errors Model’s along with the ratio and product estimators have been introduced in section "Shalabh and Tsai (2017) correlated MEs model’s characteristics". In section "Description of modified correlated MEs model and the proposed estimators", we have developed the Modified correlated MEs Model and the proposed the ratio and product estimators in this scenario. The properties of the suggested estimators are examined up to first order of approximation (foa). We have covered the bias and MSE comparisons of the suggested mean per unit, ratio and product estimators with the usual mean per unit as well as the ratio and product estimators given by Shalabh and Tsai^19^ in sections "[Sec Sec4]" and "[Sec Sec5]", respectively. The theoretical efficiency conditions of proposed estimators were also obtained. In Section "Special Case", a special case of the recommended ratio and product estimators under modified correlated MEs was also discussed.

In Section "Empirical study ", an empirical study is also provided for assessing the efficiency of proposed estimators. In section "Simulation study ", a simulation study has also been performed in R software to strengthen the current study. The results and discussion followed by conclusion of the current study are summarized in sections “Results and discussions” and “Conclusion”, respectively.

## Shalabh and Tsai (2017) correlated MEs model’s characteristics

Let $\Omega = (\Omega_{1} ,\,\Omega_{2} ,\,...,\,\Omega_{N} )$ be a finite population of size N and a sample of size n be selected from the population $\Omega$ using SRSWOR scheme. Assume that the true value of the ith unit of $\Omega$ is denoted by X_i_ and Y_i_ corresponding to the auxiliary and study variables, respectively.

But these true values are somehow not available and rather these are detected as $y_{i}$ and $x_{i}$ having MEs denoted by $u_{i}$ and $v_{i}$, respectively. Shalabh and Tsai^19^ assumed that these values can be expressed in additive form defined as:

1 $$y_{i} = Y_{i} + u_{i} ,\;\;\;x_{i} = X_{i} + v_{i} \,;\,\,\,\,i = 1,2, \ldots ,n$$

The MEs $u_{i}$ and $v_{i}$ are unobservable and assumed to have mean 0 (zero) and different variances $\sigma_{u}^{2} \,$ and $\sigma_{v}^{2}$, respectively, with correlation coefficient $\rho_{uv} .$ Moreover it is reasonable assuming uncorrelated MEs to the true values. Suppose that $\mu_{Y} \,$ and $\mu_{X}$ are the population means, $\sigma_{Y}^{2}$ and $\sigma_{X}^{2}$ are the population variances, $C_{Y}$ and $C_{X}$ are the population coefficients of variation while $\rho_{YX}$ is the population correlation coefficient. Further, consider $\overline{y} = \frac{1}{n}\sum\limits_{i = 1}^{n} {y_{i} }$ and $\overline{x} = \frac{1}{n}\,\sum\limits_{i = 1}^{n} {x_{i} }$ as the sample means of the observed values.

Assuming known population mean $\mu_{X}$ of the auxiliary variable X, Shalabh and Tsai^19^ proposed ratio as well as product estimators for the population mean $\mu_{Y}$ of the study variable Y given as:

2 $$t_{R} = \overline{y}\left( {\mu_{X} /\overline{x}} \right)$$

3 $$and\;\;t_{P} = \overline{y}\left( {\overline{x}/\mu_{X} } \right)$$

Assuming large enough population size N, the finite population correction (fpc) term is $\left( {1 - f} \right) \cong 1,\,\,and\,\,f = \frac{n}{N}$ (sampling fraction), i.e., $f = \frac{n}{N} \cong 0$.

It is easy to see that $\overline{y}$ is an unbiased estimator of $\mu_{Y}$ and its variance/mean squared error (MSE) is given as:

4 $$Var(\overline{y}) = MSE(\overline{y}) = \frac{1}{n}\left( {\sigma_{Y}^{2} + \sigma_{u}^{2} } \right)$$

5 $${\text{Similarly}},\;\;Var(\overline{x}) = MSE(\overline{x}) = \frac{1}{n}\left( {\sigma_{X}^{2} + \sigma_{v}^{2} } \right)$$

6 $$and\;\;Cov(\overline{y},\overline{x}) = \frac{1}{n}\,\left( {\rho_{YX} \sigma_{Y} \sigma_{X} + \rho_{uv} \sigma_{u} \sigma_{v} } \right)$$

The bias and MSE of $t_{R}$ and $t_{P}$ up to first order of approximation (foa), are respectively, given as:

7 $$B\left( {t_{R} } \right) = \frac{1}{n}\left( {B_{R} + B_{RM} } \right),$$

8 $$B\left( {t_{P} } \right) = \frac{1}{n}\left( {B_{P} + B_{PM} } \right),$$

9 $$MSE\left( {t_{R} } \right) = \frac{1}{n}\left( {V_{R} + V_{RM} } \right),$$

10 $$MSE(t_{P} ) = \frac{1}{n}\left( {V_{P} + V_{PM} } \right),$$

where $$B_{R} = \frac{{\sigma_{Y} \sigma_{X} }}{{\mu_{X} }}\left( {\frac{{\mu_{Y} \sigma_{X} }}{{\mu_{X} \sigma_{Y} }} - \rho_{YX} } \right) = \mu_{Y} C_{X}^{2} (1 - K_{YX} ),$$ $$B_{P} = \frac{{\rho_{YX} \sigma_{Y} \sigma_{X} }}{{\mu_{X} }} = \mu_{Y} C_{X}^{2} K_{YX} ,$$

$$B_{RM} = \frac{{\sigma_{u} \sigma_{v} }}{{\mu_{X} }}\left( {\frac{{\mu_{Y} \sigma_{v} }}{{\mu_{X} \sigma_{u} }} - \rho_{uv} } \right) = \frac{{R\sigma_{v}^{2} }}{{\mu_{X} }}\left( {1 - K_{uv} } \right),$$

$$B_{PM} = \frac{1}{{\mu_{X} }}\rho_{uv} \sigma_{u} \sigma_{v} = \frac{{R\sigma_{v}^{2} }}{{\mu_{X} }}K_{uv} ,$$

$$V_{R} = \sigma_{Y}^{2} \left[ {1 - 2\left( {\frac{{\mu_{Y} \sigma_{X} }}{{\mu_{X} \sigma_{Y} }}} \right)\rho_{YX} + \left( {\frac{{\mu_{Y} \sigma_{X} }}{{\mu_{X} \sigma_{Y} }}} \right)^{2} } \right] = \mu_{Y}^{2} \left[ {C_{Y}^{2} + C_{X}^{2} \left( {1 - 2\,K_{YX} } \right)} \right],$$

$$V_{P} = \sigma_{Y}^{2} \left[ {1 + 2\left( {\frac{{\mu_{Y} \sigma_{X} }}{{\mu_{X} \sigma_{Y} }}} \right)\rho_{YX} + \left( {\frac{{\mu_{Y} \sigma_{X} }}{{\mu_{X} \sigma_{Y} }}} \right)^{2} } \right] = \mu_{Y}^{2} \,\left[ {C_{Y}^{2} + C_{X}^{2} \left( {1 + 2\,K_{YX} } \right)} \right],$$

$$V_{RM} = \sigma_{u}^{2} \left[ {1 - 2\left( {\frac{{\mu_{Y} \sigma_{v} }}{{\mu_{X} \sigma_{u} }}} \right)\rho_{uv} + \left( {\frac{{\mu_{Y} \sigma_{v} }}{{\mu_{X} \sigma_{u} }}} \right)^{2} } \right] = \left[ {\sigma_{u}^{2} + R^{2} \sigma_{v}^{2} \left( {1 - 2\,K_{uv} } \right)} \right],$$

$$V_{PM} = \sigma_{u}^{2} \left[ {1 + 2\left( {\frac{{\mu_{Y} \sigma_{v} }}{{\mu_{X} \sigma_{u} }}} \right)\rho_{uv} + \left( {\frac{{\mu_{Y} \sigma_{v} }}{{\mu_{X} \sigma_{u} }}} \right)^{2} } \right] = \left[ {\sigma_{u}^{2} + R^{2} \sigma_{v}^{2} \left( {1 + 2K_{uv} } \right)} \right],$$

$$R = \mu_{Y} /\mu_{X} ,\,\,K_{YX} = \rho_{YX} \left( {C_{Y} /C_{X} } \right) = \beta_{YX} /R,\,\,K_{uv} = \left( {\beta_{uv} /R} \right),$$

$$\,\beta_{YX} = \rho_{YX} \left( {\sigma_{Y} /\sigma_{X} } \right),\,\,\beta_{uv} = \rho_{uv} \,\left( {\sigma_{u} /\sigma_{v} } \right).$$

## Description of modified correlated MEs model and the proposed estimators

We define the following correlated MEs model for expressing the observed values $y_{i}^{*}$ and $x_{i}^{*}$ in the additive form of true values (denoted by X_i_ and Y_i_) and the MEs (denoted by $u_{i}$ and $v_{i}$, respectively) as:

11 $$\left. \begin{gathered} y_{i}^{*} = Y_{i} + \alpha u_{i} ,\,\,\,\,\,\,\,\,\,\,\,\,\,\,\,\,\,\,\,\,\,\,\,\,\,\,\,\,\,\,\,\,\,\,\,\,\,\,\,\,\,\,\,\,\,\,\,\,\hfill \\ x_{i}^{*} = X_{i} + \eta v_{i} ;\,\,\,\,\,i = 1,\,\,2,\,\,...,\,\,n \hfill \\ \end{gathered} \right\}$$

where $\left( {\alpha ,\eta } \right)$ are constants to be determined the conditions over $\left( {\alpha ,\eta } \right)$ so that the model (11) is superior to the Shalabh and Tsai^19^ model defined in (1).

The sample means denoted by $\overline{y}^{*}$ and $\overline{x}^{*}$ under the model (11) are defined as:

$$\overline{y}^{*} = \frac{1}{n}\,\sum\limits_{i = 1}^{n} {y_{i}^{*} } \;\;and\;\;\overline{x}^{*} = \frac{1}{n}\,\sum\limits_{i = 1}^{n} {x_{i}^{*} } .$$

Estimators $\overline{y}^{*}$ and $\overline{x}^{*}$ can easily be proved as unbiased estimators of the population means $\mu_{Y}$ and $\mu_{X} ,$ respectively.

The variances/MSEs of $\left( {\overline{y}^{*} ,\,\,\overline{x}^{*} } \right)$ and the covariance between $\left( {\overline{y}^{*} \,and\,\,\overline{x}^{*} } \right)$ under SRSWOR ignoring fpc term, are respectively, given by

12 $$Var\,(\overline{y}^{*} ) = MSE(\overline{y}^{*} ) = \frac{1}{n}\,\left( {\sigma_{Y}^{2} + \alpha^{2} \sigma_{u}^{2} } \right)$$

13 $$Var\,(\overline{x}^{*} ) = MSE(\overline{x}^{*} ) = \frac{1}{n}\,\left( {\sigma_{X}^{2} + \eta^{2} \sigma_{v}^{2} } \right)$$

14 $$Cov\,\left( {\overline{y}^{*} ,\overline{x}^{*} } \right) = \frac{1}{n}\left( {\rho_{YX} \sigma_{Y} \sigma_{X} + \alpha \eta \,\,\rho_{vu} \sigma_{u} \sigma_{v} } \right)$$

From (4) and (12), we have $MSE(\overline{y}^{*} ) < MSE(\overline{y})$ if

$$(1 - \alpha^{2} ) > 0$$

15 $${\text{i}}.{\text{e}}.,{\text{ if}}\;\left| \alpha \right| < 1$$

Similarly, from (5) and (13), we note that

$$MSE(\overline{x}^{*} ) < MSE(\overline{x})\,$$ if

$$(1 - \eta^{2} ) > 0$$

16 $${\text{i}}.{\text{e}}.,{\text{ if}} \;\left| \eta \right| < 1$$

Thus, the resulting modified correlated MEs model is:

17 $$\left. \begin{gathered} y_{i}^{*} = Y_{i} + \alpha \,u_{i} ,\,\,\,\,\,\,\,\,\,\,\,\,\,\,\,\,\,\,\,\,\,\,\,\,\,\,\,\,\,\,\,\,\,\,\,\,\,\,\,\,\,\, \hfill \\ x_{i}^{*} = X_{i} + \eta v_{i} ,\,\,\,\,\,i = 1,\,2,...,n \hfill \\ \end{gathered} \right\}$$

with $$\left| \alpha \right| < 1$$ and $$\left| \eta \right| < 1$$.

Here we note that $\alpha$ and $\eta$ may take the values of $\rho_{YX}$ and $\rho_{uv}$ as $\left| {\rho_{YX} } \right| < 1$ and $\left| {\rho_{uv} } \right| < 1$.

Now we define the ratio ($t_{R}^{*}$) and product ($t_{P}^{*}$) estimators for population mean $\mu_{Y}$ of Y under the model (17) as

18 $$t_{R}^{*} = \frac{{\overline{y}^{*} }}{{\overline{x}^{*} }}\,\mu_{X}$$

19 $$and\;\;t_{P}^{*} = \frac{{\overline{y}^{*} \overline{x}^{*} }}{{\mu_{X} }}$$

To study the properties of the estimators $t_{R}^{*}$ and $t_{P}^{*}$ under the model (17), we write

$$w_{y} = \frac{1}{\sqrt n }\sum\limits_{i = 1}^{n} {(Y_{i} - \mu_{Y} ),\,\,\,w_{x} = \frac{1}{\sqrt n }\,\sum\limits_{i = 1}^{n} {(X_{i} - \mu_{X} )} } ,\;w_{u} = \frac{1}{\sqrt n }\,\sum\limits_{i = 1}^{n} {u_{i} } \;and\;w_{v} = \frac{1}{\sqrt n }\sum\limits_{i = 1}^{n} {v_{i} } .$$

20 $$Thus\;\;\overline{y}^{*} = \mu_{Y} + \frac{1}{\sqrt n }(w_{y} + \alpha w_{u} ),$$

21 $$\overline{x}^{*} = \mu_{X} + \frac{1}{\sqrt n }\left( {w_{x} + \eta w_{v} } \right),$$

We note that $e_{0}^{*} = (\overline{y}^{*} - \mu_{Y} ) = \frac{1}{\sqrt n }\,(w_{y} + \alpha w_{u} )$

$$e_{1}^{*} = (\overline{x}^{*} - \mu_{X} ) = \frac{1}{\sqrt n }\,(w_{x} + \eta w_{v} )$$

such that $E(e_{0}^{*} ) = E(e_{1}^{*} ) = 0$, $E(e_{0}^{*2} ) = \frac{1}{n}\,\left( {\sigma_{Y}^{2} + \alpha^{2} \sigma_{u}^{2} } \right)$, $E(e_{1}^{*2} ) = \frac{1}{n}\left( {\sigma_{x}^{2} + \eta^{2} \sigma_{v}^{2} } \right)$ and $E\left( {e_{0}^{*} e_{1}^{*} } \right) = \frac{1}{n}\left( {\rho_{YX} \sigma_{Y} \sigma_{X} + \alpha \eta \,\,\rho_{v\,u} \,\sigma_{u} \sigma_{v} } \right)$.

Stating $t_{R}^{*}$ in the form of $e_{0}^{*} \,$ and $e_{1}^{*}$, we have

22 $$t_{R}^{*} = \mu_{Y} \left( {1 + \frac{{e_{0}^{*} }}{{\mu_{Y} }}} \right)\,\left( {1 + \frac{{e_{1}^{*} }}{{\mu_{X} }}} \right)^{ - 1}$$

Assuming $\left| {\frac{{e_{1}^{*} }}{{\mu_{X} }}} \right| < 1$, the term $\left( {1 + \frac{{e_{1}^{*} }}{{\mu_{X} }}} \right)^{ - 1}$ will be expandable. Expanding (22) up to power two of $e^{\prime}s$, we have

$$t_{R}^{*} = \mu_{Y} \left[ {1 + \frac{{e_{0}^{*} }}{{\mu_{Y} }} - \frac{{e_{1}^{*} }}{{\mu_{X} }} + \frac{{e_{1}^{*2} }}{{\mu_{X}^{2} }} - \frac{{e_{0}^{*} e_{1}^{*} }}{{\mu_{Y} \mu_{X} }}} \right]$$

23 $$or\;\left( {t_{R}^{*} - \mu_{Y} } \right) = \mu_{Y} \left( {\frac{{e_{0}^{*} }}{{\mu_{Y} }} - \frac{{e_{1}^{*} }}{{\mu_{X} }} + \frac{{e_{1}^{*2} }}{{\mu_{X}^{2} }} - \frac{{e_{0}^{*} e_{1}^{*} }}{{\mu_{Y} \mu_{X} }}} \right)$$

We obtain the bias of $t_{R}^{*}$ up to foa by taking expectation of (23) which is given as:

24 $$B\left( {t_{R}^{*} } \right) = \frac{1}{n}\,\left( {B_{R} + B_{RM}^{*} } \right),$$

25 $${\text{where}}\;B_{RM}^{*} = \frac{{\eta \,R\sigma_{v}^{2} }}{{\mu_{X} }}\,\left( {\eta - \alpha \,K_{uv} } \right)$$

It is observed from (24) that the bias of $t_{R}^{*}$ will vanish when sample size n is sufficiently large. Further, the bias of $t_{R}^{*}$ at (24) can be re-expressed as:

$$B\left( {t_{R}^{*} } \right) = \frac{R}{{n\mu_{X} }}\,\left[ {\sigma_{x}^{2} (1 - K_{YX} ) + \eta \sigma_{v}^{2} \,(\eta - \alpha K_{uv} )} \right]$$

which will be zero, if

$$\beta _{{YX}} = R\;\;and\;\;\alpha \beta _{{uv}} = \eta R.$$

Thus, under the conditions, $\beta_{YX} = R$ and $\alpha \beta_{uv} = \eta R$, the proposed ratio estimator $t_{R}^{*}$ is almost unbiased.

After squaring (23) and ignoring greater than power two terms of $e^{*}$’s, we obtain

26 $$\left( {t_{R}^{*} - \mu_{Y} } \right)^{2} = \,\,\left( {e_{0}^{*2} - 2R\,e_{0}^{*} e_{1}^{*} + R^{2} e_{1}^{*2} } \right)$$

The expectation of (26) provides the mean squared error of $t_{R}^{*}$ up to foa given as:

27 $$MSE\left( {t_{R}^{*} } \right) = \frac{1}{n}\,\left( {V_{R} + V_{RM}^{*} } \right),$$

28 $${\text{where}}\;\;V_{RM}^{*} = \left[ {\alpha^{2} \sigma_{u}^{2} + R^{2} \,\sigma_{v}^{2} \left( {\eta^{2} - 2\alpha \eta \,K_{uv} } \right)} \right]$$

We note that if $\rho_{uv}$ is positive, then we select $(\alpha ,\eta )$ in such a way that the quantity $\alpha \eta$ is also positive. Alternatively, if $\rho_{uv}$ is negative, then we choose $\left( {\alpha ,\eta } \right)$ in such a manner that the quantity $\alpha \eta$ is negative. We also note that $\left( {\alpha ,\eta } \right)$ may also take the values of correlation coefficients $\rho_{YX}$ and $\rho_{uv}$. Progressing similar to $t_{R}^{*} ,$ the following expressions for the bias and MSE of the product estimator $t_{P}^{*}$ up to foa can be obtained as follows:

29 $$B\left( {t_{P}^{*} } \right) = E\left( {t_{P}^{*} - \mu_{Y} } \right) = \frac{1}{n}\,\left( {B_{P} + B_{PM}^{*} } \right),$$

30 $$MSE(t_{P}^{*} ) = E(t_{P}^{*} - \mu_{Y} )^{2} = \frac{1}{n}\,(V_{P} + V_{PM}^{*} ),$$

31 $${\text{where}}\;\;\;B_{PM}^{*} = \frac{{R\sigma_{v}^{2} \,}}{{\mu_{X} }}\alpha \eta \,K_{uv} ,$$

32 $$V_{PM}^{*} = \left[ {\alpha^{2} \sigma_{u}^{2} + R^{2} \sigma_{v}^{2} \,\left( {\eta^{2} + 2\alpha \eta \,K_{uv} } \right)} \right]$$

Expression (29) clearly shows that the bias of $t_{P}^{*}$ is zero, for sufficiently large n. The bias of $t_{P}^{*}$ at (29) can be re-written as:

$$B\left( {t_{P}^{*} } \right) = \frac{1}{{n\mu_{X} }}\,\left[ {\sigma_{x}^{2} \beta_{YX} + \sigma_{v}^{2} \alpha \eta K_{uv} )} \right]$$

The above expression clearly indicates that the product estimator $t_{P}^{*}$ is unbiased if $\rho_{YX} = 0$ and $\rho_{uv} = 0,$ i.e., if the correlation between the two variables Y and X is zero and the measurement error variables u and v are uncorrelated.

Further, the bias of the ratio estimator $t_{R}^{*}$ (of the product estimator $t_{P}^{*}$) decreases as the sample size n increases and can be easily seen that the proposed ratio (product) estimator $t_{R}^{*} \,(t_{P}^{*} )$ is consistent.

We note from (32) that if $\rho_{uv}$ is positive, then to get large efficiency we select $(\alpha ,\eta )$ in such a way that the quantity $\alpha \eta$ is negative. On the other hand, if $\rho_{uv}$ is negative, then we choose $\left( {\alpha ,\eta } \right)$ in such a manner that the quantity $\alpha \eta$ is positive.

It can be noticed from the expressions of bias and MSE of the estimators $(t_{R} ,t_{P} ,t_{R}^{*} ,t_{P}^{*} )$ that data having existence of the MEs lead a supplementary term in each instance. However, this additional term disappears in case of no MEs on both the variables.

This study can also be extended on the lines of Shahzad et al.^25^ and Ali et al.^26^.

## Bias comparisons of $t_{R} ,\,\,t_{R}^{*} ,\,\,t_{P}$ and $t_{P}^{*}$

It is looked upon based on the results obtain in sections "Shalabh and Tsai (2017) correlated MEs model’s characteristics" and "Description of modified correlated MEs model and the proposed estimators" that estimators $\overline{y}$ and $\overline{y}^{*}$ are unbiased whereas $t_{R} ,t_{R}^{*} ,t_{P}$ and $t_{P}^{*}$ are biased estimators of the population mean $\mu_{Y}$ of Y. This fact holds correct whether the MEs exist or do not exist.

- From (7) and (24), we have that $\left| {B(t_{R}^{*} )} \right| < \left| {B(t_{R} )} \right|$ if
  $$\left| {\eta \left( {\eta - \alpha K_{uv} } \right)\,} \right| < \left| {(1 - K_{uv} )} \right|$$
  33 $${\text{i}}.{\text{e}}.,{\text{ if}}\;\;\left[ {(1 - \eta^{4} ) + (1 - \alpha^{2} \eta^{2} )\,K_{uv}^{2} - 2K_{uv}^{{}} (1 - \alpha \eta^{3} )} \right] > 0$$

This inequality will meet usually in survey situations as $\left| \alpha \right| < 1$ and $\left| \eta \right| < 1$.

Now, we consider the two situations:

1. if $\eta = \alpha$ then the inequality (33) reduces to
  34 $$\left( {1 - \alpha^{4} } \right)\,\,(1 - K_{uv} )^{2} > 0$$
  which always holds good as $\left| \alpha \right| < 1$. Thus when $\eta = \alpha$ the proposed ratio estimator $t_{R}^{*}$ is always less biased than the Shalabh and Tsai^19^ ratio estimator $t_{R}$.
2. if $\rho_{uv} = 0$, i.e., MEs $u_{i}$ and $v_{i}$ are not correlated, then inequality (33) boils down to:
  35 $$\left( {1 - \eta^{4} } \right) > 0$$
  which again holds good as $\left| \eta \right| < 1$.

Thus in this situation $(\rho_{uv} = 0),$ the suggested estimator $t_{R}^{*}$ is less biased than the Shalabh^7^ and Shalabh and Tsai^19^ ratio estimator $t_{R} .$ Here we would like to mention that the properties of $t_{R}$ have been studied by Shalabh^7^ in case of no correlation between the MEs.

- From (8) and (29), we note that
  36 $$\begin{gathered} \left| {B(t_{P}^{*} )} \right| < \left| {B(t_{P} )} \right|\;\;{\text{if}} \hfill \\ \left| {(B_{P} + B_{PM}^{*} )} \right| < \left| {(B_{P} + B_{PM} )} \right| \hfill \\ {\text{i}}.{\text{e}}.,{\text{ if}}\left| {B_{PM}^{*} } \right| < \left| {B_{PM} } \right| \hfill \\ {\text{i}}.{\text{e}}.,{\text{ if}}\left| {\alpha \eta K_{uv} } \right| < \left| {K_{uv} } \right| \hfill \\ {\text{i}}.{\text{e}}.,{\text{ if}}\left| {\alpha \eta } \right| < 1 \end{gathered}$$

This is always true because $\left| \alpha \right| < 1$ and $\left| \eta \right| < 1$.

## Comparison of MSEs of $(t_{R}^{*} ,t_{P}^{*} )$ with $(\overline{y},\,\,\overline{y}^{*} ,\,\,t_{R} ,\,\,t_{P} )$

- From (4), (9), (12) and (27) it is noted that

1. the suggested estimator $t_{R}^{*}$ is said to be more efficient than the conventional unbiased estimator $\overline{y}$ and the suggested estimator $\overline{y}^{*}$, respectively, if
  37 $$R^{2} \left[ {\sigma_{X}^{2} (1 - 2K_{YX} ) + \sigma_{v}^{2} \eta \,(\eta - 2\alpha \,K_{uv} )} \right] < (1 - \alpha^{2} )\,\sigma_{u}^{2} ,$$
  38 $$and\;\;\left[ {\sigma_{X}^{2} (1 - 2\,K_{YX} ) + \sigma_{v}^{2} \eta \,(\eta - 2\alpha \,K_{uv} )} \right] < 0$$
2. The proposed estimator $\overline{y}^{*}$ is more precise than Shalabh and Tsai^19^ estimator $t_{R}$ if
  39 $$\left[ {R^{2} \left\{ {\sigma_{X}^{2} (1 - 2\,K_{YX} ) + \sigma_{v}^{2} (1 - 2\,K_{uv} )} \right\} + (1 - \alpha^{2} )\,\sigma_{u}^{2} } \right] > 0$$
3. the developed estimator $t_{R}^{*}$ has smaller MSE than Shalabh and Tsai^19^) estimator $t_{R}$ if
  $$V_{RM}^{*} < V_{RM}$$
  40 $${\text{i}}.{\text{e}}.,{\text{ if}} \;\left[ {\sigma_{u}^{2} (1 - \alpha^{2} ) + R^{2} \sigma_{V}^{2} \left\{ {(1 - \eta^{2} ) - 2K_{uv} (1 - \alpha \eta )} \right\}} \right] > 0$$

Now we consider the two situations:

- if $\rho_{uv} = 0$, then inequalities (37)–(40), respectively, reduce to:
  41 $$R^{2} \left[ {\sigma_{X}^{2} (1 - 2K_{YX} ) + \sigma_{v}^{2} \eta^{2} } \right] < (1 - \alpha^{2} )\,\sigma_{u}^{2}$$
  42 $$\left[ {\sigma_{X}^{2} (1 - 2\,K_{YX} ) + \sigma_{v}^{2} \eta^{2} } \right] < 0$$
  43 $$\left[ {R^{2} \left\{ {\sigma_{X}^{2} (1 - 2\,K_{YX} ) + \sigma_{v}^{2} } \right\} + \left( {1 - \alpha^{2} } \right)\,\sigma_{u}^{2} } \right] > 0$$
  44 $$\left[ {\sigma_{u}^{2} (1 - \alpha^{2} ) + R^{2} \sigma_{v}^{2} (1 - \eta^{2} )} \right] > 0$$

From (44) it is clear that when $\rho_{uv} = 0,$ the recommended ratio estimator $t_{R}^{*}$ is always better than Shalabh^7^, and Shalabh and Tsai^19^ ratio estimator $t_{R}$, as in this case the inequality (44) always holds good. If $K_{YX} < \frac{1}{2}$, then the inequality (43) holds true, i.e., the suggested estimator $\overline{y}^{*}$ is said to be more efficient than Shalabh and Tsai^19^ estimator $t_{R} ,$ while for $K_{YX} < \frac{1}{2},$ the inequality (42) does not hold good, i.e., the suggested ratio estimator $t_{R}^{*}$ is inferior to the proposed estimator $\overline{y}^{*} .$ If $K_{YX} < \frac{1}{2},$ inequality (41) is not hard to meet in the survey situations which suggests that the offered ratio estimator $t_{R}^{*}$ is better than the conventional unbiased estimator $\overline{y}$.

(b) if $\eta = \alpha$, then inequalities (37), (38) and (40), respectively, boils down to:
  45 $$R^{2} \left[ {\sigma_{X}^{2} (1 - 2\,K_{YX} ) + \sigma_{v}^{2} \eta^{2} (1 - 2K_{uv} )} \right] < (1 - \alpha^{2} )\,\sigma_{u}^{2} ,$$
  46 $$\left[ {\sigma_{X}^{2} (1 - 2\,K_{YX} ) + \sigma_{v}^{2} \eta^{2} (1 - 2\,K_{uv} )} \right] < 0,$$
  47 $$(1 - \alpha^{2} )\,\left[ {\sigma_{u}^{2} + R^{2} \sigma_{v}^{2} (1 - 2\,K_{uv} )} \right] > 0$$

It is observed from (45)–(47) that the proposed ratio estimator $t_{R}^{*}$ is more efficient than the estimator:

(i) $\overline{y}$ if the inequality (45) holds good.
(ii) $\overline{y}^{*}$, if $K_{YX} > \frac{1}{2}$ and $K_{uv} > \frac{1}{2}\,.$
(iii) Shalabh and Tsai^19^ ratio estimator $t_{R}$ as $\left| \alpha \right| < 1$.

- From (4), (10), (12) and (30), we observe that the offered product estimator $t_{P}^{*}$ is better than the estimator:
  48 $$\overline{y}\,\; {\text{if}}\;R^{2} \left[ {\sigma_{X}^{2} (1 + 2\,K_{YX} ) + \sigma_{v}^{2} \left( {\eta^{2} + 2\alpha \eta \,K_{uv} } \right)} \right] < (1 - \alpha^{2} )\,\sigma_{u}^{2}$$
  49 $$\overline{y}^{*} \;\;{\text{if}}\;\left[ {\sigma_{X}^{2} (1 + 2K_{YX} ) + \sigma_{v}^{2} \left( {\eta^{2} + 2\alpha \eta \,K_{uv} } \right)} \right] < 0$$

$t_{P}$(due to Shalabh and Tsai^19^) if

50 $$\left[ {\sigma_{u}^{2} (1 - \alpha^{2} ) + R^{2} \sigma_{v}^{2} \left\{ {(1 - \eta^{2} ) + 2(1 - \alpha \eta )\,K_{uv} } \right\}} \right] > 0$$

It is further observed from (9) and (12) that $MSE(\overline{y}^{*} ) < MSE(t_{P} )$, if

51 $$\left\{ {R^{2} \left[ {\sigma_{X}^{2} (1 + 2\,K_{YX} ) + \sigma_{v}^{2} (1 + 2\,K_{uv} )} \right] + \sigma_{u}^{2} (1 - \alpha^{2} )} \right\} > 0$$

Now we discuss two cases:

(c) if $\rho_{uv} = 0$, then conditions (48)–(51), respectively, reduce to:
  52 $$R^{2} \left[ {\sigma_{X}^{2} (1 + 2\,K_{YX} ) + \sigma_{v}^{2} \eta^{2} } \right]\, < (1 - \alpha^{2} )\,\sigma_{u}^{2} ,$$
  53 $$\left[ {\sigma_{X}^{2} (1 + 2K_{YX} ) + \eta^{2} \sigma_{v}^{2} } \right] < 0,$$
  54 $$\left[ {\sigma_{u}^{2} \left( {1 - \alpha^{2} } \right) + R^{2} \sigma_{v}^{2} (1 - \eta^{2} )} \right] > 0\,,$$
  55 $$\left\{ {R^{2} \left[ {\sigma_{X}^{2} (1 + 2\,K_{YX} ) + \sigma_{v}^{2} } \right] + \sigma_{u}^{2} (1 - \alpha^{2} )} \right\} > 0$$

Inequality (54) clearly propagates that the recommended product estimator $t_{P}^{*}$ is better than Shalabh and Tsai^19^ product estimator $t_{p}$ as $\left| \alpha \right| < 1$ and $\left| \eta \right| < 1$. Further $t_{P}^{*}$ is more efficient than $\overline{y}$ and $\overline{y}^{*}$ provided that the inequalities (52) and (53) hold, respectively. If condition (55) is satisfied, then the suggested estimator $\overline{y}^{*}$ is better than the product estimator $t_{P}$ due to Shalabh and Tsai^19^.

(d) if $\eta = \alpha$, then the inequalities (48)–(51), respectively, reduce to:
  56 $$R^{2} \left[ {\sigma_{X}^{2} (1 + 2\,K_{YX} ) + \sigma_{v}^{2} \alpha^{2} (1 + 2\,K_{uv} )} \right] < (1 - \alpha^{2} )\,\sigma_{u}^{2}$$
  57 $$\left[ {\sigma_{X}^{2} (1 + 2\,K_{YX} ) + \sigma_{v}^{2} (1 + 2\,K_{uv} )} \right] < 0$$
  58 $$(1 - \alpha^{2} )\,\left[ {\sigma_{u}^{2} + R^{2} \sigma_{v}^{2} (1 + 2\,K_{uv} )} \right] > 0$$
  59 $$\left[ {R^{2} \left\{ {\sigma_{X}^{2} (1 + 2K_{YX} ) + \sigma_{v}^{2} (1 + 2\,K_{uv} )} \right\} + \sigma_{u}^{2} (1 - \alpha^{2} )} \right] > 0$$

From (58) it follows the proposed product estimator $t_{P}^{*}$ is better than Shalabh and Tsai^19^ product estimator $t_{P}$ as long as $\left[ {\sigma_{u}^{2} + R^{2} \sigma_{v}^{2} \left( {1 + 2\,K_{uv} } \right)} \right] > 0$. The proposed product estimator $t_{P}^{*}$ will be more efficient than $\overline{y}$ and $\overline{y}^{*}$, if the conditions (56) and (57), respectively, hold good. Further the estimator $\overline{y}^{*}$ is superior to the Shalabh and Tsai^19^ product estimator $t_{P}$ as long as the inequality (59) satisfied.

(e) We now compare the estimators $t_{R}^{*}$ and $t_{P}^{*}$. From (27) and (30), we have that

$$MSE(t_{R}^{*} ) < MSE(t_{p}^{*} ),{\text{ if}}$$

$$(V_{R} + V_{RM}^{*} ) < (V_{P} + V_{PM}^{*} )$$

60 $${\text{i}}.{\text{e}}.,{\text{ if}}\,\,\,\rho_{YX} + \alpha \eta \left( {\frac{{\sigma_{u} }}{{\sigma_{Y} }}} \right)\left( {\frac{{\sigma_{v} }}{{\sigma_{X} }}} \right)\rho_{uv} > 0$$

provided that the ratio $R = \frac{{\mu_{Y} }}{{\mu_{X} }}$ is non-negative and (α, η) have the same signs.

When there are no measurement errors in the auxiliary variable and/or the measurement errors associated with the study and auxiliary variables are not correlated, the condition (60) boils down to $\rho_{YX} > 0,$ which is usual condition derived under the specification of no measurement errors.

If we set α = η = 1 in (60), then we have

61 $$\rho_{YX} + \left( {\frac{{\sigma_{u} }}{{\sigma_{Y} }}} \right)\left( {\frac{{\sigma_{v} }}{{\sigma_{X} }}} \right)\rho_{uv} > 0$$

which is due to Shalabh and Tsai^19^.

## Special case

For $\eta = \alpha ,$ we define the ratio and product estimators for $\mu_{Y}$ under modified correlated MEs, respectively, as:

62 $$t_{R}^{**} = \frac{{\overline{y}^{*} }}{{\overline{x}^{**} }}\mu_{X} ,$$

63 $$and\;\;t_{P}^{**} = \frac{{\overline{y}^{*} \overline{x}^{**} }}{{\mu_{X} }},$$

where $$\overline{y}^{*} = \left( {\overline{Y} + \alpha \overline{u}} \right)$$ and $$\overline{x}^{**} = \left( {\overline{X} + \alpha \overline{v}} \right)$$ with $$\left| \alpha \right| < 1$$.

Putting $$\eta = \alpha$$ in (24), (29), (27) and (30), we derive the bias and MSE of $$t_{R}^{**}$$ and $$t_{P}^{**}$$ up to foa, respectively, as:

64 $$B\left( {t_{R}^{**} } \right) = \frac{1}{n}\left( {B_{R} + B_{RM}^{**} } \right),$$

65 $$B\left( {t_{P}^{**} } \right) = \frac{1}{n}\left( {B_{P} + B_{PM}^{**} } \right),$$

66 $$MSE\left( {t_{R}^{**} } \right) = \frac{1}{n}\left( {V_{R} + V_{RM}^{**} } \right),$$

67 $$MSE\left( {t_{P}^{**} } \right) = \frac{1}{n}\left( {V_{P} + V_{PM}^{**} } \right),$$

where $$B_{RM}^{**} = \frac{{\alpha^{2} R\sigma_{v}^{2} }}{{\mu_{X} }}(1 - K_{uv} ),\,$$$$B_{PM}^{**} = \frac{{\alpha^{2} R\sigma_{v}^{2} }}{{\mu_{X} }}K_{uv} ,$$

$$V_{RM}^{**} = \alpha^{2} V_{RM} = \alpha^{2} \left[ {\sigma_{u}^{2} + R^{2} \sigma_{v}^{2} (1 - 2K_{uv} )} \right],\,$$ and $$V_{PM}^{**} = \alpha^{2} V_{PM} = \alpha^{2} \left[ {\sigma_{u}^{2} + R^{2} \sigma_{v}^{2} (1 + 2K_{uv} )} \right]$$.

From (7)–(10), (64)–(67), it can be easily proved that the proposed ratio (or, product) estimator $$t_{R}^{**} \,(or,\,\,t_{P}^{**} )$$ at (62) (or, (63)) is less biased as well as more efficient than Shalabh and Tsai^19^ ratio (or, product) estimator $$t_{R} (or,\,\,t_{P} )$$ at (2) (or, (3)) under the restriction $$\left| \alpha \right| < 1$$.

From (4) and (66), we have that

68 $$MSE\left( {t_{R}^{**} } \right) < MSE(\overline{y})\;\;if\;\;\alpha^{2} < \frac{{\left\{ {\sigma_{u}^{2} + R^{2} \sigma_{X}^{2} (2K_{YX} - 1)} \right\}}}{{\left\{ {\sigma_{u}^{2} + R^{2} \sigma_{v}^{2} (1 - 2\,K_{uv} )} \right\}}}$$

Further from (12) and (66), we observe that $$MSE(t_{R}^{**} ) < MSE(\overline{y}^{*} )\,$$ if

69 $$\alpha^{2} < \frac{{\sigma_{X}^{2} (2K_{YX} - 1)}}{{\sigma_{v}^{2} (1 - 2K_{uv} )}}$$

Hence, the recommended estimator $$t_{R}^{**}$$ is better than $$\overline{y}$$ and $$\overline{y}^{*}$$, respectively, if the inequalities (68) and (69) are satisfied.

From (4) and (67), it is reflected that

70 $$MSE(t_{P}^{**} ) < MSE(\overline{y})\,\,\;\;if\;\;\alpha^{2} < \frac{{\left\{ {\sigma_{u}^{2} - R^{2} \sigma_{X}^{2} (1 + 2K_{YX} )} \right\}}}{{\left\{ {\sigma_{u}^{2} + R^{2} \sigma_{v}^{2} (1 + K_{uv} )} \right\}}}$$

Further from (12) and (67), we have that

$$MSE(t_{P}^{**} ) < MSE(\overline{y}^{*} )$$ if

71 $$\alpha^{2} < - \frac{{\sigma_{X}^{2} (1 + 2K_{YX} )}}{{\sigma_{v}^{2} (1 + 2K_{uv} )}}$$

Thus the recommended product estimator $$t_{P}^{**}$$ is better than $$\overline{y}$$ and $$\overline{y}^{*}$$ provided that the inequalities (70) and (71) satisfied, respectively.

## Empirical study

To judge the performance of the recommended estimators, we have performed an empirical study using two real populations earlier considered by Bhushan et al.^21,22^.

**Population I:** Source: Gujarati and Sangeetha (2007).

$Y_{i} \,\,and\,\,y_{i}$ are true and measured consumption expenditure, respectively.

$X_{i} \,\,and\,\,x_{i}$ are true and measured income, respectively.

**Population II:** Source: The book of U.S. Census Bureau (1986).

$Y_{i} \,\,and\,\,y_{i}$ are true and measured value of the product sold, respectively.

$X_{i} \,\,and\,\,x_{i}$ are true and measured size of farms, respectively.

**Table:** 

Population	N	n	$\mu_{Y}$	$\,\mu_{X}$	$\sigma_{Y}^{2}$	$\sigma_{X}^{2}$	$\sigma_{u}^{2}$	$\sigma_{v}^{2}$	$\rho_{YX}$	$\rho_{uv}$
I	10	4	127	170	1278	3300	36	36	0.964	0.800
II	56	15	61.59	75.79	577.44	155.5	16	16	− 0.508	− 0.418

We have used the following formulae for computing percent relative efficiencies (PREs) of various estimators of $\mu_{Y}$ with respect to $\overline{y}:$

$$PRE(\overline{y}^{*} ,\,\,\overline{y}) = \frac{{\left( {\sigma_{Y}^{2} + \sigma_{u}^{2} } \right)}}{{\left( {\sigma_{Y}^{2} + \alpha^{2} \sigma_{v}^{2} } \right)}} \times 100$$, $$PRE(t_{R} ,\overline{y}) = \frac{{\left( {\sigma_{Y}^{2} + \sigma_{u}^{2} } \right)}}{{\left( {V_{R} + V_{RM} } \right)}} \times 100$$,

$$PRE\left( {t_{R}^{**} ,\overline{y}} \right) = \frac{{\left( {\sigma_{Y}^{2} + \sigma_{u}^{2} } \right)}}{{\left( {V_{R} + \alpha^{2} V_{RM} } \right)}} \times 100$$, $$PRE(t_{P} ,\overline{y}) = \frac{{\left( {\sigma_{Y}^{2} + \sigma_{u}^{2} } \right)}}{{\left( {V_{P} + V_{PM} } \right)}} \times 100$$ and 

$$PRE(t_{P}^{**} ,\overline{y}) = \frac{{\left( {\sigma_{Y}^{2} + \sigma_{u}^{2} } \right)}}{{\left( {V_{P} + \alpha^{2} V_{PM} } \right)}} \times 100$$.

These values are displayed in Tables 1, 2, 3 and 4.

**Table 1:** Biases, MSEs and PREs (with respect to $\overline{y}$) of estimators $t_{R} ,t_{P}$ for Populations I and II.

Estimator	Bias			MSE			PRE		
	$\overline{y}$	$t_{R}$	$t_{P}$	$\overline{y}$	$t_{R}$	$t_{P}$	$\overline{y}$	$t_{R}$	$t_{P}$
Population-I	–	0.711	10.111	328.5	43.719	1544.187	100.000	751.391	21.273
Population-II	–	0.262	-0.580	39.563	64.331	29.895	100.000	61.498	132.340

**Table 2:** Variances/MSEs and PREs (with respect to $\overline{y}$) of $\overline{y}^{*}$ for several values of $\alpha$.

$\alpha$	Var $(\overline{y}^{*} )$		PRE $(\overline{y}^{*} ,\overline{y})$	
	Population-I	Population-II	Population-I	Population-II
0.05	319.523	38.499	102.810	102.764
0.10	319.590	38.507	102.788	102.742
0.20	319.860	38.539	102.701	102.657
0.30	320.310	38.592	102.557	102.515
0.40	320.940	38.667	102.356	102.317
0.50	321.750	38.763	102.098	102.064
0.60	322.740	38.880	101.785	101.756
0.70	323.910	39.019	101.417	101.394
0.80	325.260	39.179	100.996	100.980
0.90	326.790	39.360	100.533	100.515
1.00	328.500	39.563	102.817	102.771
0.964	327.864	39.487	100.194	100.191
0.800	321.823	38.771	100.996	100.980
− 0.508	321.073	38.682	102.075	102.041
− 0.418	319.523	38.499	102.313	102.276

**Table 3:** Biases, MSEs and PREs (with respect to $$\overline{y}$$) of $$t_{R}^{**}$$ for several values of $$\alpha$$.

$$\alpha$$	Bias $$(t_{R}^{**} )$$		MSE $$(t_{R}^{**} )$$		PRE $$(t_{R}^{**} ,\overline{y})$$	
	Population-I	Population-II	Population-I	Population-II	Population-I	Population-II
0.05	0.714	0.245	40.462	61.842	811.877	63.974
0.10	0.714	0.245	40.486	61.861	811.386	63.955
0.20	0.714	0.246	40.584	61.936	809.428	63.877
0.30	0.714	0.247	40.748	62.060	806.184	63.749
0.40	0.714	0.248	40.976	62.235	801.688	63.570
0.50	0.713	0.249	41.270	62.460	795.979	63.341
0.60	0.713	0.251	41.629	62.734	789.111	63.064
0.70	0.713	0.254	42.054	63.059	781.146	62.740
0.80	0.712	0.256	42.543	63.433	772.153	62.369
0.90	0.712	0.259	43.099	63.857	762.208	61.955
1.00	0.711	0.262	43.719	64.331	751.392	61.498
0.964	0.712	0.261	43.488	64.155	758.668	61.668
− 0.508	0.713	0.250	41.296	62.480	795.471	63.321
− 0.418	0.714	0.248	41.024	62.272	800.747	63.533

**Table 4:** Biases, MSEs and PREs (with respect to $$\overline{y}$$) of $$t_{P}^{**}$$ for several values of $$\alpha.$$.

$$\alpha$$	Bias $${(t}_{{P}}^{{{**}}} {)}$$		MSE $${(t}_{{P}}^{{{**}}} {)}$$		PRE $${(t}_{{P}}^{{{**}}} {,\overline{y})}$$	
	Population-I	Population-II	Population-I	Population-II	Population-I	Population-II
0.05	2.911	− 0.134	1519.468	28.851	21.619	137.128
0.10	2.912	− 0.134	1519.654	28.859	21.617	137.019
0.20	2.913	− 0.134	1520.397	28.890	21.606	136.942
0.30	2.915	− 0.134	1521.636	28.942	21.589	136.694
0.40	2.918	− 0.135	1523.371	29.016	21.564	136.350
0.50	2.922	− 0.135	1525.601	29.110	21.533	135.908
0.60	2.927	− 0.136	1528.327	29.225	21.494	135.373
0.70	2.932	− 0.137	1531.549	29.361	21.449	134.746
0.80	2.938	− 0.138	1535.266	29.518	21.397	134.030
0.90	2.946	− 0.139	1539.478	29.696	21.338	133.226
1.00	2.954	− 0.140	1544.187	29.895	21.273	132.340
0.964	2.951	− 0.139	1542.435	29.821	21.298	132.669
− 0.508	2.922	− 0.135	1525.801	29.118	21.530	135.869
− 0.418	2.919	− 0.135			21.559	136.277

Computation time for the empirical study: We have noted down the computation time for the numerical study. The time taken for each value of α is 0.073 s while the total computation time is 1.022 s (= 0.073 × 14).

## Simulation study 

Following Shalabh and Tsai^19^, we conducted Monte-Carlo simulation study in R software to judge the performance of the suggested estimators. We have considered the following combinations of the parameters: n = 20 and 100; $$\mu_{X} = 20;$$ $$\mu_{Y} = 30;$$$$\sigma_{X}^{2} = 1;$$ $$\sigma_{Y}^{2} = 1;$$$$\sigma_{u}^{2} = 1;$$$$\sigma_{v}^{2} = 1;$$ α = η = 1, 0.5, 0.1, 0.05 and -0.5; $$\rho_{XY}$$  =  − 0.9, − 0.5, 0, 0.5, 0.9 and $$\rho_{uv}$$  =  − 0.9, − 0.5, 0, 0.5, 0.9. For these combinations, we followed the steps given ahead:

1. Generated data on X, Y, u, and v using four-variate normal distribution considering the mean vector $\left( {\mu_{X} , \, \mu_{Y} , \, 0, \, 0} \right)^{\prime }$ and variance–covariance matrix given as:
  $$\left( {\begin{array}{*{20}c} {\sigma_{X}^{2} } & {\rho_{XY} \sigma_{X} \sigma_{Y} } & 0 & 0 \\ {\rho_{XY} \sigma_{X} \sigma_{Y} } & {\sigma_{Y}^{2} } & 0 & 0 \\ 0 & 0 & {\sigma_{u}^{2} } & {\rho_{uv} \sigma_{u} \sigma_{v} } \\ 0 & 0 & {\rho_{uv} \sigma_{u} \sigma_{v} } & {\sigma_{v}^{2} } \\ \end{array} } \right)$$
2. Estimated the values of the suggested estimators $$(t_{R}^{**} \,and\,t_{P}^{**} )$$ as well as $$\overline{y},\,\overline{y}^{*} ,\,t_{R} \,and\,t_{P}$$ on the basis of generated data for both sample sizes.
3. Computed the values of the empirical biases and mean squared errors (MSEs) of all estimators by considering 5000 replications.

The biases and MSE of the estimators for no measurement errors case, i.e., $\sigma_{u}^{2}$  = 0 and $\sigma_{v}^{2}$  = 0 for n = 20 and 100 are noted in Table 5. The biases of all estimators are given in Tables 6 and 8 for n = 20 and 100, respectively while the MSEs of theses estimators are recorded in Tables 7 and 9 for n = 20 and 100, respectively, for various combinations of α, η, $\rho_{XY}$ and $\rho_{uv}$.

**Table 5:** Bias and MSE of the estimators for no measurement errors’ case (i.e., for $\sigma_{u}^{2}$  = 0 and $\sigma_{v}^{2}$ = 0).

$\rho_{XY}$	n = 20						n = 100					
	Bias ($$\overline{y}^{*}$$)	Bias ($$t_{R}^{**}$$)	Bias ($$t_{P}^{**}$$)	MSE ($$\overline{y}^{*}$$)	MSE ($$t_{R}^{**}$$)	MSE ($$t_{P}^{**}$$)	Bias ($$\overline{y}^{*}$$)	Bias ($$t_{R}^{**}$$)	Bias ($$t_{P}^{**}$$)	MSE ($$\overline{y}^{*}$$)	MSE ($$t_{R}^{**}$$)	MSE ($$t_{P}^{**}$$)
− 0.9	0.0080	0.0219	− 0.0021	0.0502	0.3005	0.0278	0.0027	0.0084	− 0.0023	0.0097	0.0586	0.0056
− 0.5	− 0.0028	− 0.0112	0.0095	0.0506	0.2387	0.0868	− 0.0029	− 0.0051	0.0001	0.0101	0.0462	0.0169
0	− 0.0068	− 0.0124	0.0026	0.0504	0.1608	0.1635	− 0.0029	− 0.0011	− 0.0040	0.0098	0.0310	0.0330
0.5	− 0.0090	− 0.0023	− 0.0118	0.0499	0.0877	0.2396	− 0.0021	0.0027	− 0.0062	0.0095	0.0176	0.0469
0.9	− 0.0080	0.0013	− 0.0136	0.0502	0.0278	0.2998	− 0.0027	0.0021	− 0.0067	0.0097	0.0056	0.0585

**Table 6:** Bias of the estimators for n = 20 and several values of $\alpha$ and η.

$\rho_{XY}$	$\rho_{uv}$	α = η = 1			α = η = 0.5			α = η = 0.1			α = η = 0.05			α = η = − 0.5		
		Bias ($$\overline{y}$$)	Bias ($$t_{R}$$)	Bias ($$t_{P}$$)	Bias ($$\overline{y}^{*}$$)	Bias ($$t_{R}^{**}$$)	Bias ($$t_{P}^{**}$$)	Bias ($$\overline{y}^{*}$$)	Bias ($$t_{R}^{**}$$)	Bias ($$t_{P}^{**}$$)	Bias ($$\overline{y}^{*}$$)	Bias ($$t_{R}^{**}$$)	Bias ($$t_{P}^{**}$$)	Bias ($$\overline{y}^{*}$$)	Bias ($$t_{R}^{**}$$)	Bias ($$t_{P}^{**}$$)
− 0.9	− 0.9	− 0.0016	0.0145	− 0.0102	− 0.0050	0.0017	− 0.0071	− 0.0078	− 0.0063	− 0.0055	− 0.0081	− 0.0072	− 0.0053	− 0.0119	− 0.0148	− 0.0044
	− 0.5	0.0101	0.0420	− 0.0142	0.0072	0.0316	− 0.0126	0.0048	0.0251	− 0.0117	0.0045	0.0244	− 0.0116	0.0013	0.0184	− 0.0111
	0	0.0105	0.0324	− 0.0038	0.0073	0.0271	− 0.0077	0.0048	0.0243	− 0.0108	0.0045	0.0240	− 0.0112	0.0011	0.0223	− 0.0154
	0.5	0.0091	0.0224	0.0034	0.0067	0.0225	− 0.0044	0.0047	0.0234	− 0.0102	0.0045	0.0236	− 0.0109	0.0018	0.0263	− 0.0181
	0.9	− 0.0153	− 0.0037	− 0.0193	− 0.0119	− 0.0063	− 0.0128	− 0.0091	− 0.0077	− 0.0068	− 0.0088	− 0.0079	− 0.0060	− 0.0050	− 0.0090	0.0036
− 0.5	− 0.9	0.0101	0.0420	− 0.0142	0.0080	0.0286	− 0.0078	0.0063	0.0201	− 0.0036	0.0061	0.0191	− 0.0031	0.0038	0.0109	0.0014
	− 0.5	− 0.0053	0.0113	− 0.0144	− 0.0026	0.0150	− 0.0155	− 0.0005	0.0197	− 0.0169	− 0.0002	0.0204	− 0.0171	0.0027	0.0298	− 0.0197
	0	− 0.0050	0.0015	− 0.0039	− 0.0081	− 0.0037	− 0.0078	− 0.0106	− 0.0065	− 0.0109	− 0.0109	− 0.0067	− 0.0113	− 0.0143	− 0.0083	− 0.0156
	0.5	− 0.0063	0.0254	− 0.0305	− 0.0031	0.0226	− 0.0242	− 0.0006	0.0213	− 0.0187	− 0.0003	0.0213	− 0.0180	0.0032	0.0210	− 0.0098
	0.9	0.0017	0.0290	− 0.0181	0.0038	0.0232	− 0.0109	0.0055	0.0192	− 0.0044	0.0057	0.0187	− 0.0035	0.0080	0.0140	0.0068
0	− 0.9	0.0052	0.0192	− 0.0012	0.0031	0.0058	0.0052	0.0014	− 0.0028	0.0094	0.0012	− 0.0037	0.0099	− 0.0011	− 0.0120	0.0144
	− 0.5	− 0.0102	− 0.0116	− 0.0013	− 0.0046	− 0.0094	0.0048	− 0.0001	− 0.0058	0.0093	0.0005	− 0.0052	0.0098	0.0066	0.0027	0.0153
	0	− 0.0058	0.0083	− 0.0124	− 0.0024	0.0136	− 0.0137	0.0003	0.0192	− 0.0148	0.0007	0.0200	− 0.0149	0.0044	0.0298	− 0.0163
	0.5	0.0005	0.0142	− 0.0057	0.0008	0.0042	0.0020	0.0010	− 0.0030	0.0087	0.0010	− 0.0038	0.0095	0.0013	− 0.0121	0.0193
	0.9	− 0.0032	0.0062	− 0.0050	− 0.0011	0.0004	0.0021	0.0006	− 0.0037	0.0086	0.0008	− 0.0041	0.0095	0.0031	− 0.0089	0.0198
0.5	− 0.9	0.0091	0.0224	0.0034	0.0070	0.0090	0.0098	0.0053	0.0004	0.0140	0.0051	− 0.0005	0.0145	0.0028	− 0.0088	0.0191
	− 0.5	0.0054	0.0032	0.0150	0.0081	0.0069	0.0139	0.0102	0.0117	0.0125	0.0105	0.0124	0.0123	0.0134	0.0219	0.0097
	0	0.0057	0.0273	− 0.0083	0.0026	0.0221	− 0.0122	0.0001	0.0193	− 0.0153	− 0.0002	0.0190	− 0.0157	− 0.0036	0.0174	− 0.0200
	0.5	0.0044	0.0174	− 0.0011	0.0076	0.0146	0.0052	0.0101	0.0133	0.0107	0.0104	0.0132	0.0114	0.0139	0.0129	0.0196
	0.9	0.0007	0.0094	− 0.0004	0.0028	0.0036	0.0067	0.0045	− 0.0005	0.0132	0.0047	− 0.0010	0.0141	0.0070	− 0.0057	0.0244
0.9	− 0.9	0.0153	0.0269	0.0112	0.0119	0.0142	0.0143	0.0091	0.0061	0.0159	0.0088	0.0053	0.0161	0.0050	− 0.0023	0.0170
	− 0.5	0.0017	0.0290	− 0.0181	− 0.0013	0.0186	− 0.0164	− 0.0036	0.0121	− 0.0156	− 0.0039	0.0114	− 0.0155	− 0.0072	0.0054	− 0.0150
	0	0.0020	0.0194	− 0.0076	− 0.0011	0.0141	− 0.0115	− 0.0036	0.0113	− 0.0147	− 0.0039	0.0110	− 0.0151	− 0.0073	0.0094	− 0.0193
	0.5	0.0007	0.0094	− 0.0004	− 0.0018	0.0094	− 0.0082	− 0.0037	0.0104	− 0.0140	− 0.0040	0.0106	− 0.0147	− 0.0067	0.0134	− 0.0220
	0.9	0.0016	0.0087	0.0021	0.0050	0.0062	0.0086	0.0078	0.0047	0.0146	0.0081	0.0046	0.0154	0.0119	0.0034	0.0250

**Table 7:** MSE of the estimators for n = 20 and several values of $\alpha$ and η.

	$\rho_{uv}$	α = η = 1			α = η = 0.5			α = η = 0.1			α = η = 0.05			α = η = − 0.5		
$$\rho_{xy}$$		MSE ($$\overline{y}$$)	MSE ($$t_{R}$$)	MSE ($$t_{P}$$)	MSE ($$\overline{y}^{*}$$)	MSE ($$t_{R}^{**}$$)	MSE ($$t_{P}^{**}$$)	MSE ($$\overline{y}^{*}$$)	MSE ($$t_{R}^{**}$$)	MSE ($$t_{P}^{**}$$)	MSE ($$\overline{y}^{*}$$)	MSE ($$t_{R}^{**}$$)	MSE ($$t_{P}^{**}$$)	MSE ($$\overline{y}^{*}$$)	MSE ($$t_{R}^{**}$$)	MSE ($$t_{P}^{**}$$)
− 0.9	− 0.9	0.1008	0.6026	0.0555	0.0628	0.3737	0.0342	0.0504	0.2993	0.0274	0.0500	0.2967	0.0272	0.0620	0.3679	0.0343
	− 0.5	0.0994	0.5357	0.1172	0.0624	0.3582	0.0497	0.0508	0.3023	0.0282	0.0505	0.3007	0.0275	0.0636	0.3613	0.0498
	0	0.0994	0.4687	0.1935	0.0625	0.3428	0.0689	0.0508	0.3021	0.0290	0.0505	0.3008	0.0277	0.0633	0.3410	0.0686
	0.5	0.1005	0.3946	0.2647	0.0629	0.3248	0.0867	0.0509	0.3015	0.0297	0.0506	0.3007	0.0279	0.0630	0.3211	0.0862
	0.9	0.0992	0.3258	0.3273	0.0620	0.3036	0.1022	0.0502	0.2961	0.0301	0.0499	0.2958	0.0279	0.0628	0.3021	0.1020
− 0.5	− 0.9	0.0994	0.5357	0.1172	0.0622	0.3112	0.0967	0.0506	0.2401	0.0901	0.0503	0.2380	0.0899	0.0635	0.3137	0.0967
	− 0.5	0.1009	0.4805	0.1752	0.0624	0.2990	0.1085	0.0501	0.2402	0.0873	0.0497	0.2383	0.0867	0.0622	0.2959	0.1093
	0	0.1002	0.4040	0.2500	0.0626	0.2803	0.1269	0.0504	0.2412	0.0881	0.0500	0.2400	0.0870	0.0620	0.2820	0.1295
	0.5	0.1003	0.3275	0.3238	0.0624	0.2597	0.1453	0.0501	0.2383	0.0887	0.0497	0.2377	0.0870	0.0619	0.2608	0.1476
	0.9	0.1020	0.2647	0.3915	0.0635	0.2442	0.1658	0.0509	0.2376	0.0931	0.0505	0.2374	0.0907	0.0622	0.2442	0.1637
0	− 0.9	0.1008	0.4648	0.1915	0.0636	0.2388	0.1708	0.0519	0.1663	0.1641	0.0516	0.1640	0.1639	0.0647	0.2377	0.1703
	− 0.5	0.1019	0.4023	0.2480	0.0643	0.2228	0.1842	0.0521	0.1656	0.1644	0.0517	0.1638	0.1639	0.0638	0.2235	0.1867
	0	0.1029	0.3263	0.3244	0.0646	0.2049	0.2020	0.0521	0.1658	0.1629	0.0517	0.1645	0.1616	0.0636	0.2041	0.2022
	0.5	0.1031	0.2508	0.4069	0.0647	0.1853	0.2256	0.0522	0.1642	0.1666	0.0517	0.1635	0.1646	0.0635	0.1844	0.2214
	0.9	0.1031	0.1913	0.4683	0.0647	0.1704	0.2411	0.0522	0.1636	0.1673	0.0517	0.1633	0.1648	0.0636	0.1698	0.2362
0.5	− 0.9	0.1005	0.3946	0.2647	0.0627	0.1670	0.2439	0.0507	0.0933	0.2372	0.0503	0.0908	0.2370	0.0629	0.1626	0.2434
	− 0.5	0.1019	0.3259	0.3254	0.0631	0.1463	0.2586	0.0504	0.0888	0.2374	0.0499	0.0870	0.2368	0.0620	0.1461	0.2593
	0	0.1009	0.2519	0.4093	0.0632	0.1275	0.2826	0.0509	0.0880	0.2409	0.0505	0.0868	0.2395	0.0624	0.1284	0.2779
	0.5	0.1006	0.1760	0.4795	0.0626	0.1084	0.2981	0.0503	0.0871	0.2393	0.0499	0.0865	0.2373	0.0620	0.1096	0.2950
	0.9	0.1007	0.1176	0.5408	0.0629	0.0969	0.3139	0.0507	0.0902	0.2403	0.0503	0.0899	0.2378	0.0627	0.0965	0.3096
0.9	− 0.9	0.0992	0.3285	0.3259	0.0620	0.1025	0.3041	0.0502	0.0302	0.2969	0.0499	0.0279	0.2966	0.0628	0.1020	0.3031
	− 0.5	0.1020	0.2647	0.3915	0.0636	0.0864	0.3230	0.0511	0.0296	0.3005	0.0506	0.0279	0.2998	0.0624	0.0868	0.3208
	0	0.1010	0.1933	0.4707	0.0633	0.0687	0.3436	0.0510	0.0289	0.3016	0.0506	0.0277	0.3001	0.0625	0.0689	0.3381
	0.5	0.1007	0.1176	0.5408	0.0630	0.0499	0.3609	0.0509	0.0282	0.3023	0.0506	0.0275	0.3003	0.0629	0.0498	0.3561
	0.9	0.1008	0.0555	0.6006	0.0628	0.0342	0.3736	0.0504	0.0274	0.2999	0.0500	0.0272	0.2975	0.0620	0.0343	0.3695

**Table 8:** Bias of the estimators for n = 100 and several values of $\alpha$ and η.

$\rho_{xy}$	$\rho_{uv}$	α = η = 1			α = η = 0.5			α = η = 0.1			α = η = 0.05			α = η = − 0.5		
		Bias ($$\overline{y}$$)	Bias ($$t_{R}$$)	Bias ($$t_{P}$$)	Bias ($$\overline{y}^{*}$$)	Bias ($$t_{R}^{**}$$)	Bias ($$t_{P}^{**}$$)	Bias ($$\overline{y}^{*}$$)	Bias ($$t_{R}^{**}$$)	Bias ($$t_{P}^{**}$$)	Bias ($$\overline{y}^{*}$$)	Bias ($$t_{R}^{**}$$)	Bias ($$t_{P}^{**}$$)	Bias ($$\overline{y}^{*}$$)	Bias ($$t_{R}^{**}$$)	Bias ($$t_{P}^{**}$$)
− 0.9	− 0.9	0.0036	0.0187	− 0.0069	0.0024	0.0098	− 0.0034	0.0014	0.0040	− 0.0005	0.0012	0.0034	− 0.0002	− 0.0001	− 0.0023	0.0037
	− 0.5	0.0019	0.0178	− 0.0096	0.0025	0.0120	− 0.0052	0.0031	0.0087	− 0.0017	0.0032	0.0084	− 0.0013	0.0039	0.0060	0.0035
	0	0.0025	0.0096	− 0.0001	0.0029	0.0079	− 0.0005	0.0031	0.0079	− 0.0008	0.0032	0.0080	− 0.0008	0.0036	0.0102	− 0.0013
	0.5	0.0033	0.0030	0.0080	0.0033	0.0046	0.0036	0.0032	0.0072	0.0000	0.0032	0.0076	− 0.0004	0.0032	0.0134	− 0.0054
	0.9	− 0.0014	0.0136	− 0.0119	− 0.0001	0.0073	− 0.0059	0.0009	0.0035	− 0.0010	0.0010	0.0032	− 0.0004	0.0024	0.0002	0.0062
− 0.5	− 0.9	0.0019	0.0202	− 0.0120	0.0003	0.0090	− 0.0068	− 0.0010	0.0014	− 0.0027	− 0.0012	0.0006	− 0.0022	− 0.0030	− 0.0077	0.0034
	− 0.5	− 0.0030	− 0.0100	0.0085	− 0.0014	− 0.0065	0.0054	− 0.0001	− 0.0023	0.0029	0.0001	− 0.0017	0.0026	0.0018	0.0063	− 0.0009
	0	− 0.0024	− 0.0041	0.0039	− 0.0020	− 0.0059	0.0036	− 0.0017	− 0.0059	0.0033	− 0.0017	− 0.0059	0.0032	− 0.0013	− 0.0037	0.0028
	0.5	− 0.0016	0.0090	− 0.0076	− 0.0007	0.0030	− 0.0027	0.0000	− 0.0004	0.0013	0.0001	− 0.0007	0.0018	0.0011	− 0.0032	0.0072
	0.9	− 0.0046	0.0137	− 0.0184	− 0.0030	0.0058	− 0.0101	− 0.0017	0.0008	− 0.0034	− 0.0015	0.0002	− 0.0025	0.0003	− 0.0045	0.0067
0	− 0.9	0.0018	0.0210	− 0.0129	0.0002	0.0097	− 0.0077	− 0.0011	0.0021	− 0.0036	− 0.0013	0.0013	− 0.0031	− 0.0030	− 0.0070	0.0026
	− 0.5	− 0.0031	0.0018	− 0.0034	− 0.0023	0.0001	− 0.0030	− 0.0016	0.0002	− 0.0026	− 0.0015	0.0003	− 0.0026	− 0.0006	0.0026	− 0.0021
	0	− 0.0043	− 0.0120	0.0077	− 0.0029	− 0.0086	0.0044	− 0.0017	− 0.0045	0.0018	− 0.0016	− 0.0039	0.0015	0.0000	0.0038	− 0.0020
	0.5	− 0.0048	0.0175	− 0.0227	− 0.0031	0.0080	− 0.0126	− 0.0018	0.0018	− 0.0046	− 0.0016	0.0011	− 0.0036	0.0003	− 0.0054	0.0076
	0.9	− 0.0047	0.0144	− 0.0192	− 0.0030	0.0065	− 0.0109	− 0.0018	0.0015	− 0.0042	− 0.0016	0.0009	− 0.0034	0.0002	− 0.0038	0.0058
0.5	− 0.9	0.0033	0.0232	− 0.0121	0.0017	0.0119	− 0.0069	0.0004	0.0043	− 0.0028	0.0002	0.0035	− 0.0022	− 0.0015	− 0.0048	0.0034
	− 0.5	− 0.0048	− 0.0104	0.0054	− 0.0031	− 0.0068	0.0022	− 0.0018	− 0.0026	− 0.0003	− 0.0017	− 0.0020	− 0.0006	0.0001	0.0060	− 0.0041
	0	− 0.0041	0.0011	− 0.0048	− 0.0038	− 0.0006	− 0.0052	− 0.0035	− 0.0006	− 0.0055	− 0.0034	− 0.0005	− 0.0056	− 0.0030	0.0017	− 0.0060
	0.5	− 0.0033	0.0086	− 0.0107	− 0.0024	0.0027	− 0.0058	− 0.0017	− 0.0007	− 0.0019	− 0.0016	− 0.0011	− 0.0014	− 0.0006	− 0.0035	0.0040
	0.9	− 0.0032	0.0166	− 0.0184	− 0.0015	0.0087	− 0.0101	− 0.0003	0.0037	− 0.0034	− 0.0001	0.0031	− 0.0026	0.0017	− 0.0016	0.0066
0.9	− 0.9	0.0014	0.0156	− 0.0083	0.0001	0.0067	− 0.0047	− 0.0009	0.0009	− 0.0019	− 0.0010	0.0003	− 0.0015	− 0.0024	− 0.0054	0.0023
	− 0.5	− 0.0046	0.0105	− 0.0152	− 0.0039	0.0047	− 0.0108	− 0.0034	0.0014	− 0.0073	− 0.0033	0.0010	− 0.0069	− 0.0025	− 0.0013	− 0.0021
	0	− 0.0040	0.0023	− 0.0057	− 0.0036	0.0005	− 0.0061	− 0.0033	0.0005	− 0.0064	− 0.0033	0.0006	− 0.0064	− 0.0029	0.0029	− 0.0068
	0.5	− 0.0032	− 0.0043	0.0025	− 0.0032	− 0.0027	− 0.0020	− 0.0032	− 0.0001	− 0.0056	− 0.0032	0.0003	− 0.0060	− 0.0033	0.0061	− 0.0109
	0.9	− 0.0036	0.0105	− 0.0132	− 0.0024	0.0041	− 0.0072	− 0.0014	0.0004	− 0.0024	− 0.0012	0.0000	− 0.0018	0.0001	− 0.0029	0.0049

**Table 9:** MSE of the estimators for n = 100 and several values of $\alpha$ and η.

$\rho_{xy}$	$\rho_{uv}$	α = η = 1			α = η = 0.5			α = η = 0.1			α = η = 0.05			α = η = —0.5		
		MSE ($$\overline{y}$$)	MSE ($$t_{R}$$)	MSE ($$t_{P}$$)	MSE ($$\overline{y}^{*}$$)	MSE ($$t_{R}^{**}$$)	MSE ($$t_{P}^{**}$$)	MSE ($$\overline{y}^{*}$$)	MSE ($$t_{R}^{**}$$)	MSE ($$t_{P}^{**}$$)	MSE ($$\overline{y}^{*}$$)	MSE ($$t_{R}^{**}$$)	MSE ($$t_{P}^{**}$$)	MSE ($$\overline{y}^{*}$$)	MSE ($$t_{R}^{**}$$)	MSE ($$t_{P}^{**}$$)
− 0.9	− 0.9	0.0202	0.1860	0.1262	0.0127	0.0907	0.0353	0.0101	0.0600	0.0065	0.0100	0.0590	0.0056	0.0121	0.0895	0.0363
	− 0.5	0.0190	0.1788	0.1288	0.0119	0.0876	0.0365	0.0098	0.0593	0.0068	0.0098	0.0586	0.0058	0.0127	0.0910	0.0358
	0	0.0189	0.1817	0.1297	0.0119	0.0883	0.0363	0.0098	0.0593	0.0066	0.0098	0.0586	0.0057	0.0126	0.0920	0.0371
	0.5	0.0191	0.1811	0.1259	0.0120	0.0888	0.0352	0.0098	0.0595	0.0066	0.0098	0.0587	0.0057	0.0125	0.0902	0.0366
	0.9	0.0192	0.1791	0.1307	0.0121	0.0883	0.0366	0.0100	0.0597	0.0066	0.0100	0.0589	0.0057	0.0127	0.0899	0.0369
− 0.5	− 0.9	0.0190	0.1708	0.1376	0.0121	0.0773	0.0473	0.0100	0.0479	0.0185	0.0100	0.0470	0.0176	0.0128	0.0787	0.0474
	− 0.5	0.0204	0.1746	0.1388	0.0125	0.0796	0.0480	0.0099	0.0486	0.0188	0.0098	0.0475	0.0179	0.0119	0.0769	0.0476
	0	0.0201	0.1760	0.1406	0.0125	0.0795	0.0477	0.0100	0.0480	0.0184	0.0099	0.0469	0.0175	0.0122	0.0768	0.0494
	0.5	0.0199	0.1719	0.1421	0.0124	0.0785	0.0483	0.0099	0.0484	0.0187	0.0098	0.0474	0.0178	0.0119	0.0773	0.0499
	0.9	0.0206	0.1688	0.1424	0.0128	0.0776	0.0486	0.0102	0.0481	0.0185	0.0101	0.0471	0.0176	0.0121	0.0761	0.0486
0	− 0.9	0.0195	0.1583	0.1515	0.0123	0.0636	0.0620	0.0101	0.0331	0.0336	0.0100	0.0321	0.0328	0.0126	0.0627	0.0634
	− 0.5	0.0201	0.1558	0.1543	0.0126	0.0627	0.0629	0.0101	0.0330	0.0338	0.0100	0.0321	0.0329	0.0123	0.0631	0.0634
	0	0.0201	0.1493	0.1523	0.0126	0.0606	0.0618	0.0101	0.0332	0.0333	0.0100	0.0325	0.0325	0.0123	0.0649	0.0641
	0.5	0.0200	0.1512	0.1574	0.0125	0.0614	0.0640	0.0101	0.0329	0.0339	0.0100	0.0320	0.0329	0.0123	0.0622	0.0632
	0.9	0.0200	0.1517	0.1590	0.0126	0.0615	0.0645	0.0101	0.0329	0.0340	0.0100	0.0321	0.0330	0.0123	0.0623	0.0632
0.5	− 0.9	0.0191	0.1439	0.1657	0.0120	0.0493	0.0763	0.0098	0.0189	0.0481	0.0098	0.0179	0.0472	0.0125	0.0486	0.0779
	− 0.5	0.0206	0.1420	0.1679	0.0128	0.0483	0.0771	0.0102	0.0184	0.0479	0.0101	0.0175	0.0470	0.0123	0.0487	0.0767
	0	0.0204	0.1429	0.1711	0.0126	0.0484	0.0776	0.0100	0.0186	0.0478	0.0099	0.0177	0.0469	0.0119	0.0498	0.0781
	0.5	0.0201	0.1386	0.1727	0.0126	0.0470	0.0782	0.0102	0.0182	0.0479	0.0101	0.0174	0.0470	0.0123	0.0490	0.0782
	0.9	0.0201	0.1376	0.1737	0.0125	0.0474	0.0791	0.0099	0.0187	0.0484	0.0098	0.0179	0.0474	0.0120	0.0480	0.0775
0.9	− 0.9	0.0192	0.1310	0.1788	0.0121	0.0366	0.0882	0.0100	0.0066	0.0597	0.0100	0.0056	0.0588	0.0127	0.0369	0.0899
	− 0.5	0.0206	0.1294	0.1809	0.0127	0.0365	0.0890	0.0100	0.0067	0.0596	0.0098	0.0058	0.0586	0.0119	0.0361	0.0890
	0	0.0204	0.1312	0.1830	0.0126	0.0366	0.0894	0.0100	0.0066	0.0595	0.0098	0.0057	0.0586	0.0119	0.0377	0.0897
	0.5	0.0201	0.1278	0.1809	0.0125	0.0357	0.0892	0.0099	0.0066	0.0596	0.0098	0.0057	0.0587	0.0120	0.0373	0.0884
	0.9	0.0202	0.1263	0.1855	0.0127	0.0353	0.0906	0.0101	0.0065	0.0600	0.0100	0.0056	0.0590	0.0121	0.0363	0.0894

Computation time for the simuation study: We have noted down the computation time for the simulation study also. The time taken for one iteration (for each combination of α, $\rho_{xy}$ and $\rho_{uv}$) is 2.138 s while the total computation time is 4.632333 min (= 2.138 × (5 × 5 × 5 + 5))/60).

## Results and discussions

From Tables 1, 2, 3 and 4, we observe the followings:

1. The proposed ratio estimator $$t_{R}^{**}$$ has bias very marginally larger than the ratio estimator $$t_{R}$$ in population-I while it (proposed ratio estimator $$t_{R}^{**}$$) is less biased than the ratio estimator $$t_{R}$$ for the population-II. Further, it is observed that the suggested product estimator $$t_{P}^{**}$$ is less biased (in the sense of absolute bias) than the product estimator $$t_{P}$$ for both the populations I and II.
2. The recommended unbiased estimator $\overline{y}^{*}$ is more efficient than the conventional unbiased estimator $\overline{y}$ with marginal gain in efficiency for $\left| \alpha \right| < 1$ in Populations I and II.
3. The recommended ratio estimator $$t_{R}^{**}$$ is more efficient than $$\overline{y},\,\,\overline{y}^{*} ,\,t_{P}$$ and Shalabh and Tsai^19^ ratio estimator $$t_{R}$$ with considerable gain in efficiency under the condition $$\left| \alpha \right| < 1$$ in Population I, while it is inferior to $$\overline{y}$$ and $$\overline{y}^{*}$$ in Population II due to negative correlation between $$\left( {Y\& X} \right)$$ and $$(u_{i} \, \& \, v_{i} )$$ but $$t_{R}^{**}$$ is superior to Shalabh and Tsai^19^ product estimator $$t_{P}$$.
4. The recommended product estimator $$t_{P}^{**}$$ is better than the estimators $$\overline{y},\,\,\overline{y}^{*}$$ and Shalabh and Tsai^19^ product estimator $$t_{P}$$ under the condition $$\left| \alpha \right| < 1$$ in Population II, while it is inferior to the estimators $$\overline{y}$$ and $$\overline{y}^{*}$$ in Population I due to positive correlation between $$\left( {Y\& X} \right)$$ and $$\left( {u_{i} \, \& \, v_{i} } \right)$$ but superior to $$t_{P}$$ with very marginal gain in efficiency. It happened due to moderate correlation between $$(X\& Y)$$ and $$(u_{i} \, \& \, v_{i} )$$ in Population I.

Similarly, from Tables 5, 6, 7, 8 and 9, we can compare the biases and MSEs under both conditions without measurement error as well as in the presence of measurement error. From these Tables 5, 6, 7, 8 and 9, we note the followings:

1. Tables 5, 6, 7, 8, 9 clearly reveal the higher values of bias and variance or MSE under presence of measurement errors, i.e., $\sigma_{u}^{2} = 1\,\,and\, \, \sigma_{v}^{2} = 1$ than the values under no measurement errors, i.e., $\sigma_{u}^{2} = 0\,\,and\, \, \sigma_{v}^{2} = 0$. Thus it indicates that the properties of estimators got affected by the presence of measurement errors.
2. The proposed unbiased estimator $\overline{y}^{*}$ is having less bias and MSE than the conventional unbiased estimator $\overline{y}$ for $\left| \alpha \right| < 1$ and both sample sizes, i.e., n = 20, 100.
3. From Tables 5 and 7, the bias of the suggested estimators $$t_{R}^{**}$$ and $$t_{P}^{**}$$ are compared in the presence of measurement errors. It can be clearly observed that bias of $$t_{R}^{**}$$ and $$t_{P}^{**}$$ are impacted by the value of ρ_uv_ and these are substantially different for ρ_uv_ = 0 and ρ_uv_ =  ± 0.9, indicating the significant impact of correlated measurement errors. Apparently, the bias decreases as sample size increases but there is no apparent reduction in the differences in the values of bias for ρ_uv_ = 0 and ρ_uv_ =  ± 0.9. So, we can conclude that the correlated measurement errors influence the bias of the estimators compared to uncorrelated measurement errors.
4. From Tables 6 and 8, we can observe a clear impact of sign of correlation between measurement errors on the MSE values of estimators $$t_{R}^{**}$$ and $$t_{P}^{**}$$. The MSE of $$t_{R}^{**}$$ (in case of highly positively correlated study and auxiliary variable, i.e., ρ_XY_ = 0.9) is lowest for positively correlated measurement error, i.e. for ρ_uv_ = 0.9. The MSE of $$t_{R}^{**}$$ decreases As the degree of ρ_XY_ increases for ρ_XY_ > 0. However, the extent of ρ_uv_ also affects the rate and value of MSE. Obviously the MSE decreases as sample size increases for all the values of the parameters considered for ρ_XY_ > 0.
5. In the same way, we can conclude for the estimator $$t_{P}^{**}$$ (in case of highly negatively correlated study and auxiliary variable, i.e., ρ_XY_ = – 0.9) is lowest for negatively correlated measurement error, i.e. for ρ_uv_ = – 0.9. The MSE of $$t_{P}^{**}$$ decreases As the degree of ρ_XY_ increases for ρ_XY_ < 0. This clearly indicates that the presence of measurement errors affected the MSE of $$t_{R}^{**}$$ and $$t_{P}^{**}$$.
6. Tables 5, 6, 7, 8 clearly depicts that the biases and MSEs of the suggested estimators are the lowest at α = η = 0.05.

Thus, the recommended ratio $$(t_{R}^{**} )$$ and product $$(t_{P}^{**} )$$ estimators are useful in practice.

## Conclusion

This paper has introduced a modified correlated MEs model. The proposed correlated MEs model involves a constant $\alpha$ (say) with restriction $\left| \alpha \right| < 1,$ termed as ‘error control parameter’. This error control parameter $\alpha$ (say) controls the errors in observations if we choose error control parameter $\alpha$ (say) near to ‘zero’. For $\alpha = 1,$ proposed correlated MEs model reduces to Shalabh and Tsai^19^ model. We have suggested ratio as well as product estimators for population mean ($\mu_{Y}$) of the study variable Y in presence of auxiliary variable X when correlated MEs contaminate the observations on both study and auxiliary variables.

The expressions of bias and MSE of the recommended ratio and product estimators are determined up to foa under SRSWOR sampling scheme. The realistic conditions are derived under which the recommended ratio and product estimators act superior than the conventional unbiased estimators $(\overline{y},\,\,\overline{y}^{*} )$ and Shalabh and Tsai^19^ ratio $(t_{R} )$ and product $(t_{P} )$ estimators. An empirical study and a simulation study have also been performed in R software to exhibit the performance of the recommended ratio and product estimators over usual unbiased estimators and the ratio and product estimators due to Shalabh and Tsai^19^. It is observed that when the ‘error control parameter’ is close to ‘zero’, the recommended ratio and product estimators yield larger gain in efficiency. Thus, we recommend the proposed study for its use in practice.

## Data Availability

All the necessary data analyzed during the current study are included in this article.
